# Organizational culture as a mediator of credible leadership influence on work engagement: empirical studies in private hospitals in East Java, Indonesia

**DOI:** 10.1057/s41599-022-01289-z

**Published:** 2022-08-17

**Authors:** Veronika Agustini Srimulyani, Yustinus Budi Hermanto

**Affiliations:** 1 Widya Mandala Surabaya Catholic University, Surabaya, East Java Indonesia; 2 Darma Cendika Catholic University, Surabaya, East Java Indonesia

**Keywords:** Cultural and media studies, Cultural and media studies

## Abstract

The success of health services in hospitals depends on the work engagement of medical and non-medical personnel in providing quality services for patients. Engaged employees will be more proactive, take the initiative to collaborate with others, and are committed to achieving high-quality performance. A leader’s ability in an organization is a major factor in building a work ethic in the organization, instilling values, norms, and ethics through organizational culture into employee work behavior, such as work engagement. Credibility leadership is the practice of leading an organization through a positive culture change. Credible leadership can increase work engagement through an organizational culture emphasized by education and organizational leaders. The study aimed to analyze the influence of leadership credibility and organizational culture on work engagement by sampling medical and non-medical personnel at two private hospitals in Madiun City. The hypothesis was tested using structural equation modeling (SEM) and path analysis. The results of hypothesis testing show that: (1) credible leadership has a significant positive influence on organizational culture; (2) organizational culture has a significant positive influence on work engagement; (3) organizational culture is the perfect mediator of credible leadership influences on work engagement. This study’s results confirm that leaders’ role in shaping a positive organizational culture through good credible leadership practices, while organizational culture can increase employee work engagement.

## Introduction

Human resources involved in health services in a hospital are multi-professional, both health and non-health workers. Hospitals need engaged and productive employees to provide effective, efficient, fair treatment services without compromising the quality of service. Highly engaged employees are generally more productive, enjoy their work, more efficient, and are heavily involved (Tims et al., 2011). Work engagement is the driving force behind the competitiveness and success of the organization. An employee with high involvement shows a willingness to make extra efforts in work to achieve optimal performance (Engelbrecht et al., 2017). Due to the coronavirus pandemic, work engagement may experience a decline. This situation may affect the organization’s productivity (Ahmed et al., 2020); therefore, the management should take the initiative to keep employees engaged effectively, even though some work from home. The organization should support the employees to feel the effects of work and organizational engagement (Saks, 2006). Therefore, if leaders want to build a culture of subordinate engagement, they must allocate considerable time and effort to achieve the desired level of engagement (Al Shehri et al., 2017).

Hospitals in any country play a crucial role in a health system. To improve services in hospitals and win the competition in health services, hospital organizations need to develop human resources (Puspita et al., 2020). Human resources (HR) who have optimal work engagement are needed to achieve organizational goals, including in health organizations such as hospitals. Establishing high work engagement requires organizational management knowledge of the factors driving work engagement. Several ways to establish hospital employee work engagement are through leadership style and organizational culture. Leadership style and organizational culture are the two main pillars of any organization for essential organizational development. Leadership is a strategic component that can influence the traits and behaviors of organization members (Arifin et al., 2014). Leaders act as the ‘sense-givers’ and the ‘providers’ of culture, who can create the values, beliefs, and conceptions they believe are necessary and appropriate for the organization. Leadership practices in an organization play a significant role in influencing the work engagement of subordinates. The study by Gemeda and Lee (2020) showed a positive and significant influence of leadership style on work engagement and innovative behavior. How the leader establishes relationships with his subordinates, how the leader can reward outstanding subordinates, and how the leader develops and empowers his subordinates significantly affects the performance of his or her subordinates. Quality leadership is often illustrated as a reflection of organizational success or failure (Kawiana Ig et al., 2020). Several studies show that leadership impacts engagement by building trust, support, and psychological safety (The Australian Psychological Society, 2020). Credibility leadership is one of the leadership practices that can encourage the emergence of work engagement. Credibility leadership leads any organization through positive change, especially how leaders build trust, respect, and affinity with followers (Siambi, 2022). The characteristics of a credible leader, such as being honest, forward-looking, inspiring, and competent, have consistently been chosen as the four most essential leadership requirements over the last two decades (Kouzes and Posner, 2010). When a leader is trustworthy, people are more likely to commit and provide time, energy, intelligence, and support.

The leadership style that encourages employees to work with passion, enthusiasm, and focus is the visionary leadership style rather than transactional (Bass, 1999). The ability of leadership to communicate effectively creates a basis for employee engagement (Vizzuso, 2015). Influential leaders positively impact employee retention and engagement, and capable leaders positively impact productivity and performance (Matthews, 2008). The results of the studies of Hosseini et al. (2020) and Gholamzadeh et al. (2014) show a significant positive influence of leadership style (transformational and transactional leadership) on organizational culture. Leadership style indirectly impacts employee engagement through work culture as a mediation (Bija, 2020). While other studies on spiritual leadership values such as integrity, honesty, and simplicity can affect organizational culture (Alimudin et al., 2017); furthermore, organizational culture positively impacts employee engagement and effectiveness (Soni, 2019). Yang et al. (2020) show that ethical leadership affects work engagement at Chi Mei Medical Center and Chi Mei Hospital, Liouying, Taiwan. Other studies indicated that ethical leadership also positively and significantly influences work engagement (Wibawa and Takahashi, 2021), organizational culture, transformational leadership, and personal characteristics (Arifin et al., 2014).

Leaders in organizations are role models who support organizational goals and encourage employee commitment to the organization’s goals and vision. Transformational leaders change their culture by realigning organizational culture with a new vision (Bass and Avolio, 1994; Bass, 2000). Management scientists believe that leadership is a critical factor for the success of organizations and society and can affect organizational culture. Leaders influence organizational culture by defining values, beliefs, and assumptions about how things should be done and socializing those amongst the group. The leadership skills of managers and supervisors are essential in creating and strengthening cultural norms. Leadership style (transformational and transactional leadership) positively influences organizational culture and indirectly influences learning organizations with organizational culture mediating (Hosseini et al., 2020).

Another factor that directly influences work engagement is organizational culture. Organizational culture is important in building employee engagement (Hasan et al., 2020; Kalia and Verma, 2017). Organizational culture and work engagement were investigated based on the assumption that different types of culture affect employee work engagement differently. An efficient work culture helps employees feel empowered and satisfied with the work environment, thereby making employees feel involved in their work (Kalia and Verma, 2017). The organizational culture can foster employees’ work engagement (Krog, 2014). According to Al Shehri et al. (2017), when employees feel they are deriving benefits from their employer, they feel responsible for returning the favors through their work ethic. In a practical context, organizational culture was the main predictor of work engagement (Arifin et al., 2014). Several research results show the effect of organizational culture on employee engagement (Hasan et al., 2020).

Most published empirical studies are on transformational leadership and the quality of care assessing leadership styles in nursing. As the development of the results, this study focuses on how credible leadership influences organizational culture and work engagement. Credible leadership is needed to help the company develop and grow. Credible leaders influence others to be involved in achieving meaningful common goals. The influence of a credible leader is based on a solid foundation of trust and understanding, which encourages individuals to commit to the goal emotionally and exert the effort needed to achieve it, often at some personal sacrifice. Credible leaders who espouse the desired culture and reinforce or correct the behavior of others (instrumental conditioning) provide normative and social influence, which contribute to group conformity. Work engagement is selected based on studies at other hospitals in Indonesia, which state those hospital organizations need a high level of work engagement to provide the best service for patients.

This research studied the assessment of organizational members on leadership practices that have not been widely studied by previous researchers, namely credible leadership and the impact of organizational culture and work engagement. The object of the study is on medical and non-medical personnel at two private hospitals in Madiun City, East Java, Indonesia. Both hospitals are developing rapidly. Those hospitals are Siti Aisyah Islamic Hospital and St. Clara Hospital. An empirical study of credible leadership needs to be carried out to see the impact of organizational culture on increasing work engagement in the two largest hospitals in Madiun City. These two private hospitals belong to the group of 7 hospitals in the Madiun area with the best ratings from Google reviews (https://www.fakultaskedokteran.id/2022/08/keperawatan-universitas-katolik-widya-mandala-surabaya). This study also aims to analyze the indirect influence of credible leadership on work engagement through organizational culture as a mediator. The formulation of these research problems is as follows:

1. Does credible leadership influence positively and significantly the culture of the organization?
2. Does organizational culture influence positively and significantly work engagement?
3. Does credible leadership significantly influence work engagement with organizational culture as a mediator?

## Theoretical framework and hypothesis development

### Credible leadership

The role of the leader of every organization is to influence the spirit, passion in work, security, quality of work, and organizational performance and has a role in encouraging individuals and groups to achieve organizational goals. Credible leadership is the ability of a leader to influence subordinates because they can be trusted. These credible leadership qualities include (Quist, 2009):

1) demonstrated competence in leading the organization through turbulence;

2) honorable intentions in the eyes of his people;

3) commitment to personal and staff learning;

4) a leader who is comfortable dealing with people and cultures different from his own;

5) future-oriented leaders who study the driving forces of the present, looking for possible futures that the organization may experience;

6) a leader with a personal sense of creativity and innovation and the capability and commitment to providing an organizational environment conducive to creativity and innovation.

Leaders who embrace morals will respect value integrity and are trustworthy, caring, honest, and fair (Engelbrecht et al., 2017). A credible leader is not a “super” leader, a credible leader who genuinely cares about other people, possesses knowledge and skills, as well as a heart that holds high moral values for people and personal integrity (Quist, 2009). Kouzes and Posner’s theory suggests four basic characteristics of a credible leader: honest, inspiring, competent, and forward-looking or visionary (Hemby, 2017). The primary foundation of today’s leadership is credibility, honesty, competence, inspiring ability, and one new characteristic of forward-looking.

According to Matthews (2008), eight credible leader behaviors contribute to an increase in work engagement, which is briefly described as follows: First, creating a culture that reflects the values that employees can identify in a way that encourages engagement and rewards employee engagement contributions. Second, build trust through effective communication and show that employees are valuable. Third, the effective implementation of organizational strategies by making strategic changes by understanding their complexity, and after the strategies are articulated and mutually agreed upon. Fourth, communicate strategies to employees by delivering clear and consistent messages by connecting each employee with the company’s strategy and encouraging engagement, employee commitment, and work productivity. Fifth, put the strategy in the works; identify employee problems and obstacles in implementing the strategy, prioritize the human resource management strategy and align it with the company’s strategic goals. Sixth, step up and be accountable; credible leaders act with character and integrity by holding themselves accountable and behaving in a way that is consistent with the organization’s values. Seventh, manage change effectively; credible leaders must take on a more significant role to ensure change is communicated and implemented effectively. Eighth, develop leaders at all levels; credible leaders invest in their continuous learning while developing other leaders by preparing clear leadership paths for candidates who are ready now or in the future, by ensuring that they can perform at the highest level and lead effectively through change.

### Organizational culture

Organizational culture can be viewed as knowledge, habits, behaviors, values, and those made by the organization to be obeyed and carried out by all organization members in achieving organizational goals (Hasan et al., 2020). Organizational culture connects the organization’s norms, beliefs, values, and principles with its employees in a set of standards and work behavior (Soni, 2019). According to Stone et al. (2007), organizational culture is agreed upon as values and behaviors that contribute to the social and psychological environment of the organization. Complexity and a comprehensive understanding of organizational culture are essential and inevitable (Hermawan and Loo, 2019).

Organizational culture has four dimensions (Denison and Mishra, 1995):

1) Involvement, a dimension of organizational culture that shows the level of participation of organizational members in decision making. An effective organization empowers members of the organization, builds the organization, and develops the capabilities of employees at all levels;

2) Consistency, which shows the degree of agreement of the organization’s members to the organization’s basic assumptions and values. Organizations also tend to be effective because organizations have a “strong” culture, which is very consistent, coordinated, and well-integrated;

3) Adaptability is the organization’s ability to respond to changes in the external environment by making changes in the internal organization;

4) Mission is a core dimension that demonstrates the core objectives of the organization that make the members of the organization confident and steadfast in what the organization considers important. A successful organization has clear goals and directions that define organizational goals and strategic goals and reveal a vision of how the organization will look in the future.

The result of a study from Chang and Lu (2007) on Taiwanese organizations identified four characteristics of organizational culture related to stressors, namely: (1) family kin; (2) Informal work obligations; (3) organizational loyalty; and (4) subgroup involvement, where these four cultural characteristics can be interpreted as variant aspects of social support. The study by Coffey et al. (2011) showed that construction companies in Indonesia have a market culture that shows that people are very competitive and result-oriented, while clan culture companies show people share values, cohesiveness, and well-being and make the organization a pleasant place to work. Mushtaque and Siddiqui (2020) state that organizations in Pakistan must make efforts to switch to the culture of the clan with the highest value and the lowest level of work engagement and stress levels. Clan culture believes that the organization’s trust in employees and commitment toward employees allows open communication and increases employee engagement. According to Chatab (Octaviani and Fakhri, 2016), other characteristics of organizational culture in Indonesia include

1) *Integrity*, dedication in carrying out tasks, honesty in carrying out duties, maintaining honor and good name, and adherence to the applicable organizational code of ethics and regulations.;

2) *Professionalism*, which is responsible for carrying out tasks, effectiveness in carrying out tasks, discipline in carrying out tasks, and being oriented towards the future in anticipating developments, challenges and opportunities;

3) *Praiseworthy*, providing consistent role models, acting reasonably, being firm, and having a big heart;

4) *Respect for human resources* includes recruiting, developing, and retaining quality employees, treating employees based on trust, openness, fairness, and mutual respect, developing an attitude of cooperation and partnership and providing rewards based on individual and group work.

### Work engagement

Employees with a positive attitude to work tend to show more attachment to the outcome than those indifferent (Inegbedion, 2022). Attachment is one of the work attitudes in employees with high work engagement. Many people think that work and employee engagement have the same meaning but differentiating factors. The differentiating factor between work engagement and employee engagement is seen from the motivational target. For work engagement, motivation is increased by the company to its employees by establishing a positive work environment and building good communication relationships between fellow employees and others. Work engagement is work motivation built by themselves; employees are highly committed to the goals, vision, mission, and company values. The strong motivation of every employee is not only to get a high salary but to provide the best performance and results for the company. Despite having differentiating factors, both have a strong involvement. Of course, good work engagement must be balanced with employee management. If the two are mutually sustainable, it will positively impact its development.

The definition of engagement is a positive, fulfilling, work-related state of mind characterized by vigor, dedication, and absorption (Schaufeli, 2013). Kahn found three psychological conditions associated with engagement or disengagement at work: meaningfulness, safety, and availability (Arun Kumar, 2013). Work engagement is a positive and affective state of high energy combined with a high level of dedication and a strong focus on work (Schaufeli and Bakker, 2000). Three characteristics of work engagement (Schaufeli, 2013) are: (1) *Vigor* is a strong outpouring of energy and mentality during work, the courage to try hard to complete a job, and persevere in the face of work difficulties as well as the willingness to invest everything, including an effort in a job, and persistence in the face of adversity; (2) *Dedication* refers to a sense of meaning in one’s work, feeling enthusiastic and proud of the work they do, and feeling inspired and challenged by their work role; (3) *Absorption*, refers that one is completely and happily immersed in one’s work and there is difficulty disengaging from it so that time passes quickly and one forgets everything around him. Being fully concentrated and happy when involved in work causes time to feel fast even if an employee is facing problems at work. So, work engagement is characterized by a high level of individual energy and a strong identification with the work of the individual.

### The influence of credible leadership on organizational culture

Leadership effectiveness is one of the essential foundations for building a great organizational culture. Leadership effectiveness has a basis on credibility and authenticity. Both aspects can support the level of trust between leaders and their followers. Those aspects are based on the followers’ belief in the abilities and authenticity of their leaders. Organizational culture is an integral part of an organization, and a leader’s thoughts, feelings, values, and actions are influenced by it. An organization’s core values begin with its leadership, which will then develop into a leadership style (Tsai, 2011). A leader can have influence or authority in setting the tone for the culture in the organization. Leaders can help shape, develop, and preserve the desired organizational culture by creating a new set of shared values influencing organizational innovation (Al Ariss and Guo, 2016).

Leaders may significantly impact culture (Alsaqqa and Akyürek, 2021). Credible leadership can significantly affect the company’s culture. Credible leaders can create a culture of engagement based on trust. Credible leadership is the foundation for demonstrating employee trust and, in turn, inspiring employees for employee trust in leaders (Matthews, 2008). Credible leaders as an effective leadership practice in the VUCA era (volatile, uncertain, complex, and ambiguous) by strengthening organizational values by helping their employees grow and develop through positive changes in behavior and work to achieve organizational goals that are full of competition, seize the right opportunities, and recognition of the achievement of organizational goals. Previous studies show that leadership styles (transformational and transactional) significantly affect organizational culture (Hosseini et al., 2020; Gholamzadeh et al., 2014). Cultural transformation in the organizational environment can be carried out by formulating and mapping strategic values through five main behaviors as guidelines (Paramita and Kartika, 2020): (1) integrity, characterized by objective, fair, and consistent behavior; (2) professionalism, characterized by responsible behavior based on competence to achieve the best performance; (3) synergy, a collaboration of internal and external stakeholders in a productive and quality manner; (4) inclusive, open attitude and accepting the diversity of stakeholders as well as expanding public opportunities in the financial industry; (5) visionary, has broad insight and can look ahead and think outside the box. Credible leaders focus on efforts to restore subordinates by developing credibility and authenticity (Siambi, 2022). The study by Alsaqqa and Akyürek (2021) on government-owned and non-government-owned hospitals in the Gaza Strip showed a positive and significant relationship between transformational leadership style with the organizational culture of all hospitals studied. The results of other empirical studies with school objects (Karada and Öztekin, 2018) show that authentic leadership significantly impacts school culture in terms of managerial and school aims. In this study, it is predicted that the practice of credible leadership can have a positive impact on organizational culture, so the first hypothesis is formulated as follows:

H1: Credible leadership has a significant positive influence on organizational culture.

### The influence of organizational culture on work engagement

Developing a culture conducive to work engagement becomes very important for optimizing organizational performance (Martins, 2019). Psychological antecedent drivers of engagement are social support, transformational leadership, workplace climate, leader-member exchange, organizational justice, and job security (Siddique, 2019). Work engagement is measured using three dimensions, namely:

- Vigor reflects the high mental strength and mental resilience at work (optimal energy, courage to make efforts, desire, and willingness to try hard to provide maximum results in the work given).
- Dedication reflects the enthusiasm of employees at work, who take pride in their work and remain inspired by the company without feeling threatened by the challenges they face.
- Absorption reflects the condition of employees who feel happy that they are entirely immersed in their work, very concentrated, and serious about doing their job.

Organizational culture is one of the main factors for increasing work engagement. A study by Moore (2020) shows a significant impact of work culture on work engagement. In another empirical study (Bija, 2020), work culture also mediates the influence of leadership on work engagement. Nurcholis and Budi (2019) show that both work culture and perceived organizational support can increase employee engagement, either partially or simultaneously. An empirical study at nine government bank branch offices in Malang, Indonesia (Hasan et al., 2020) showed that organizational culture significantly and positively affects employee engagement. When the organizational culture matches employees’ expectations, employee engagement will be high, and vice versa. When the culture in the organization does not match employees’ expectations, employee engagement will be low. This is reinforced by another finding that the stronger the internalized organizational culture in employees, the higher the work engagement (Siddique, 2019). In this study, it was predicted that aspects of organizational culture that emphasize integrity, professionalism, exemplary, and adequate respect for human resources could increase work engagement, so the second hypothesis is formulated as follows:

H2: Organizational culture has a significant positive influence on work engagement.

### The influence of credible leadership on work engagement through organizational culture as a mediator

Leadership and organizational culture are interdependent because every aspect of leadership practice eventually shapes organizational culture. Organizational culture influences members of the organization on how to work on everything in the organization. That is, the organization’s culture affects the attitudes and behavior of all members. A strong culture can give members the coercion or coercion to act or behave as expected by the organization’s leader. A strong culture can encourage the members to act or behave as expected by the organization’s leader. A strong culture will result in good employee work behavior. For example, leadership rules are required to be disciplined on time, the company culture is disciplined, and employees always come to the office on time at work. A survey of nearly 30,000 employees in 15 countries on various topics related to organizational effectiveness, including leadership and engagement, shows statistically significant relationships and the practices and behaviors of credible leaders that appear to have the most impact on engagement (Matthews, 2008). Sürücü and Yeşilada (2017) showed that charismatic leadership positively affects organizational culture; and in other studies, organizational culture has a significant positive impact on work engagement (Martins, 2019; Soni, 2019; Siddique, 2019). Other empirical studies (Ashfaq et al., 2021) showed that ethical leadership facilitates the involvement of followers’ work directly or indirectly through self-efficacy as a mediator. Bija (2020) showed that leadership style indirectly impacts employee engagement through work culture as mediation. Yuliastuti and Tandio (2020) also mentioned that organizational culture partially mediates between charismatic leadership style with good corporate governance. Furthermore, organizational culture mediates the complete relationship between transformational leadership styles and good corporate governance. From some of the results of these empirical studies, it can be indicated that organizational culture can play a role as a mediator of the influence of credible leadership on work engagement, so the third hypothesis is formulated as follows:

H3: Credible leadership influences work engagement through organizational culture as a mediator.

**Fig. 1:**
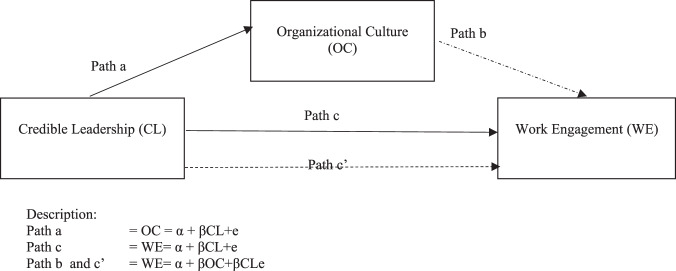
The research model.

In summary, the testing of the three hypotheses in this study is shown in Fig. 1 (research model).

Figure 1 shows the path analysis used to test the direct influence of credible leadership on organizational culture (path a) and work engagement (path c), as well as testing the indirect influence of credible leadership on work engagement through organizational culture (path b and path c’)

## Method

### Data collection

This study used a quantitative approach and was causal research to analyze credible leadership’s direct and indirect effects on work engagement with organizational culture as a mediator. The research was conducted in two private hospitals in the city of Madiun with the same type, namely type C, Siti Aisyah Islamic Hospital and Santa Clara Hospital, which received the best service assessment from hospital service users, and are currently developing facilities and infrastructure. The type of hospital is determined in four aspects: services, human resources, equipment, buildings, and infrastructure owned by the hospital. Class C type hospitals can provide limited subspecialty medical services; there are four types of specialist services: internal medicine services, surgical services, pediatric health services, and obstetrics and gynecology services. The sampling method used was quota sampling. This sampling technique takes as many samples as the number that the researcher has determined by taking into account the status of employees non-managerial (full-timer workers) and the proportion of the number of medical personnel and non-medical personnel who have the status of full-time workers in the hospitals (these are the object of research). Santa Clara Hospital ranked second out of the seven best hospitals in the Madiun area, with a 4.0/5.0 out of 166 reviews on Google. In contrast, Siti Aisyah Islamic Hospital got fourth place with a 3.9/5.0 from 443 reviews on Google (https://www.fakultaskedokteran.id/2022/08/keperawatan-universitas-katolik-widya-mandala-surabaya).

The sample at St. Aisyah Islamic Hospital was set at 185 respondents consisting of 110 medical personnel and 75 non-medical personnel, while St. Clara Hospital with 100 respondents consisting of 75 medical personnel and 25 non-medical personnel. The data collection technique was carried out by surveys using questionnaires, where the hospital’s human resources department facilitated the distribution. Measurement of credible leadership and organizational culture variables using the Likert scale ranging from strongly disagree (1), disagree (2), moderate/neutral (3), agree (4), and strongly agree (5). The measurement of the work engagement variable uses scales: never (1), sometimes (2), often (3), often (4), and always (5). Hypothesis testing using SEM analysis using the Lisrel 8.70 program and path analysis using the Sobel test.

### Measurements

Four dimensions of credible leadership are integrity, authority, power, capability (managerial), and visionary, which are developed into 35 statement items. These four dimensions refer to the theory of Kouzes and Posner (Hemby, 2017). Credible leadership was obtained from assessing medical and non-medical personnel who were respondents to the credible leadership practices of their direct supervisors. The organizational culture variable assesses respondents’ medical and non-medical personnel, measured by four dimensions: integrity, professionalism, praiseworthy, and respect for human resources, which was developed into 13 items of statement. Work engagement was measured by 17 statement items from the Utrecht work engagement scale (UWES), developed by Schaufeli and Bakker (2000), which consists of three dimensions of work engagement: vigor, dedication, and absorption.

## Result and discussion

### Result

#### Respondents characteristics

The characteristics of respondents in Table 1 include gender and group of employees. The data collection was carried out by distributing questionnaires to medical and non-medical personnel at Siti Aisyah Islamic Hospital, 185 questionnaires, and 100 questionnaires at Santa Clara Hospital, according to the number of samples that have been determined. The distributed questionnaires could not be processed by 30% or 10.52%, so this study used as many as 255 questionnaires or 89.48%.

**Table 1:** Respondents characteristics.

Category	Number of respondents	Percentage
*Gender*		
Male	63	24.7
Female	192	75.29
*Group of employees*		
Medical	161	63.14
Non-medical	94	36.86

Source: Author’s calculations.

Table 1 shows that most respondents are female (75.29%), and 24.7% are male. When viewed from the group of employees, it can be seen that most of the respondents are medical personnel (63.14%) and non-medical personnel, as much as 36.86%.

#### Variable description

The description of variables presented in Table 2 aims to provide an overview of the average answers to the overall answers of respondents to the measurement statement items of each variable, namely credible leadership, organizational culture, and work engagement of medical and non-medical personnel in two burgeoning private hospitals in Madiun City, namely Siti Aisyah Islamic Hospital and Santa Clara Hospital.

**Table 2:** Descriptive variables with average values.

No	Variables	Mean	Category
1	Credible Leadership (CL)	3.83	High
2	Organizational Culture (BO)	3.97	High
3	Work Engagement (WE)	4.18	High

Source: Author’s calculations.

Table 2 shows that the average value of credible leadership is 3.83 (high). This means that the practice of credible leadership in the hospital has received a positive response from medical and non-medical personnel. Leaders treat employees with honesty, respect, and good behavior, supervise employees fairly while doing their jobs, and hospital leaders carry out their duties in line with the hospital’s vision and mission. Furthermore, leaders also share information openly about plans, decisions, plans, and activities in the hospital so that all employees can be involved and carry out their work as well as possible. The average value of organizational culture is 3.97 (high). This indicates that a culture of honesty in carrying out their respective roles, collaborating with colleagues in fulfilling their duties, respecting each other and acting fairly in carrying out their duties, and hospital management pays attention to and appreciates the contribution of human resources has been internalized in daily work life in the work environment. Work engagement has an average value of 4.18 (very high). This means that as HR in a hospital, there is a solid sense of engagement in the work they are doing, enthusiasm, feeling inspired, proud, and challenged, which is balanced with high levels of energy and mental toughness at work, as well as the desire to try, employees will have strong resilience in the face of adversity. In the characteristics of respondents (Table 1), it is shown that the majority of respondents are women (75%), with more medical personnel than non-medical personnel (63.14%); this is indicated to be related to a high average work engagement value (4.18). Health care professionals are engaged in their work to be more resilient, dedicated, and absorbed, low experience levels of fatigue, and are healthier (van den Berg et al., 2017). Overall, it indicates that health and non-health workers are committed to their work and organization, proud of their work, willing to devote their time and energy, and have passion and high attachment to motivate themselves to contribute more to their role.

#### Construct validity test and overall model fit test

Confirmatory factor analysis (CFA) and Lisrel 8.70 software are used to test the construct validity. The test results can be seen in Table 3.

**Table 3:** Construct validity test results.

No.	Variables	Indicators of each latent variable	Loading value factor	Note
1	Credible leadership	Honesty and integrity	0.99	Valid
		Authority and power	0.55	Valid
		Capability	0.76	Valid
		Visionary	0.75	Valid
2	Organizational culture	Integrity	0.53	Valid
		Professionalism	0.67	Valid
		Praiseworthy	0.73	Valid
		Recognition for human resource	0.87	Valid
3	Work engagement	Vigor	0.96	Valid
		Dedication	0.56	Valid
		Absorption	0.62	Valid

Source: Author’s calculations.

To perform the convergent validity test (Table 3), the value of each indicator’s loading factors against its latent variables will be viewed; if these values are higher than 0.5, then the indicators are considered valid. The next stage is to analyze the suitability of the data with the Goodness of Fit (GOF) absolute fit measures. The test results can be seen in Table 4.

**Table 4:** The goodness of fit absolute fit measures.

Test	Index	Value	Result
Chi-Square	Χ^2^ > *α* = 0.05	168.71 (*p* = 0.00)	Good fit
GFI	GFI > 0.90 good fit; 0.80 ≤ GFI ≤ 0.90 marginal fit	0.89	Marginal fit
RMR	≤0.05	0.026	Good fit
RMSEA	≤0.08	0.000	Good fit
ECVI	Values that are small and close to ECVI saturated = 0.68	0.52	Good fit
NNFI	NNFI > 0.90 good fit; 80 ≤ NNFI ≤ 0.90 marginal fit	0.87	Marginal fit
NFI	NFI > 0.90 good fit; 0.80 ≤ NFI ≤ 0.90 marginal fit	0.87	Marginal fit
AGFI	AGFI > 0.90 good fit; 0.80 ≤ AGFI ≤ 0.90 marginal fit	0.89	Marginal fit
RFI	RFI > 0.90 good fit; 0.80 ≤ RFI ≤ 0.90 marginal fit	0.83	Marginal fit
IFI	IFI > 0.90 good fit; 0.80 ≤ IFI ≤ 0.90 marginal fit	0.90	Marginal fit
CFI	CFI > 0.90 good fit; 0.80 ≤ CFI ≤ 0.90 marginal fit	0.90	Marginal fit
PGFI	0.60–0.90	0.55	Marginal fit
PNFI	0.60–0.90	0.65	Good fit

Source: Author’s calculations.

A fit model can be seen from the values of Chi-Square, RMSEA, CFI, and RMR (Hooper et al., 2008). Of the 4 criteria, 3 tests (Chi-square, RMSEA, and RMR) meet the good fit criteria. The GOF test is acceptable because the results met the marginal and good fit criteria.

#### Hypothesis test

**Fig. 2:**
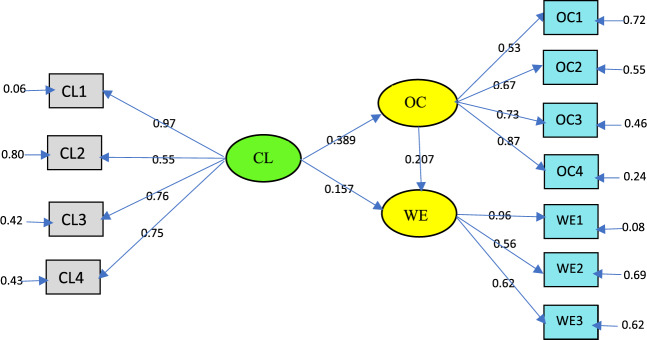
The test results of the direct influence of CL on OC and WE.

The results of path analysis for hypothesis testing can be seen in Fig. 2, Tables 5 and 6.

**Table 5:** Direct influence testing results.

Structural equations	Standardized coefficients	*t*-count	*P* value	*t*-table	Note
CL → OC (path a)	0.389	5.671	0.000	1.652	Significant
CL → WE (path c)	0.157	1.816	0.014	1.652	Significant
CL → WE (path c’)	0.055	0.843	0.400	1.652	Insignificant
OC → WE (path b)	0.173	2.642	0.009	1.652	Significant

Source: Author’s calculations.

**Table 6:** The results of the mediation influence test (Sobel test).

Path	Test statistic	Std. error	*P*-value	Note
CL → OC → WE	2.401	0.033	0.016	Significant

Source: Author’s calculations.

Testing the influence of credible leadership on work engagement through organizational culture as mediating, statistically using the Sobel test (http://quantpsy.org/sobel.htm.), summarized in Table 6. The general guidelines in this study refer to, a variable can be considered a mediator to the extent to which the independent variable carries the influence of mediating variables, and mediating variables significantly affect dependent variables. Table 5 shows that credible leadership as an independent variable significantly affects the organizational culture variable as a mediating variable (path a), and organizational culture significantly affects the work engagement variable as a dependent variable (path b).

From Table 5, the results of the influence testing are shown as follows: (1) path a indicates the partial effect of CL on OC is significantly positive; (2) path b indicates the significant positive Influence of OC on WE; (3) path c indicates the partial effect of LC on WE is significant positive; (4) path c’ indicates the effect of CL on WE is insignificant at the time OC is included in the equation. Credible leadership, as measured by honesty and integrity, capability or managerial and visionary has a significant contribution to organizational culture in terms of integrity, professionalism, praiseworthy, and recognition for human resources. *T*-count indicates this 5.670 > *t* table 1.652 with a *P*-value of 0.000; thus, H1 is accepted, namely credible leadership has a significant positive influence on organizational culture. The effect of organizational culture on work engagement showed significant positive results, with a *t*-count of 2.642 > *t* table 1.652 *P*-value of 0.009; thus, H2 is accepted; namely, organizational culture has a significant positive influence on work engagement.

In the indirect test, credible leadership on work engagement through organizational culture requires credible leadership as an independent variable that significantly influences organizational culture as mediating and organizational culture significantly influences work engagement. This refers to the provision of a variable mediator: (a) variations in the levels of the independent variable significantly account for variations in the presumed mediator (i.e., path a), (b) variations in the mediator significantly account for variations in the dependent variable (i.e., path b), and (c) when paths a and b are controlled, a previously significant effect of the independent on dependent variables is no longer significant, with the strongest demonstration of mediation occurring when path c is zero”. The results of testing with the Sobel test showed that credible indirect leadership in work engagement through organizational justice as mediating proved significant with a Sobel test value of 2.401 > *t* table 1.652 and a *P*-value of 0.016 < 0.05. In this test, it was shown that organizational culture acts as perfect mediation. This refers to, who stated that perfect mediation occurs when there is insignificant influence on the independent variable on the dependent when the mediator variable is entered in the equation; however, if the influence of the independent variable on the dependent decreases but is not equal to zero by entering the mediator, then partial mediation occurs. Path c in Table 5 shows that CL significantly affects WE before the OC variable as mediation is included in the regression equation (*t* value 1.816 > 1 *t* table 1.653). However, after the mediation variable (OC) was included in the regression equation, the effect of CL on WE decreased and was not significant (path c’) shown from the value of *t* 0.843 < *t* of table 1.653. This means H3 accepts that OC perfects the relationship between CL and WE.

## Discussion

### Influence of credible leadership on organizational culture

Test results show that credible leadership measured from the dimensions of honesty and integrity, capability (managerial), and vision significantly affect organizational culture. Credible leadership is one of the essential foundations for building a great organizational culture. A credible leader can be anyone who has influence or authority, and a leader sets the tone for organizational culture. Credible leaders can reinforce values while simultaneously holding people accountable. In HR management, leaders are advised to maintain harmony, conflict avoidance, and space to manage Indonesian staff with respect (Hermawan and Loo, 2019). Leadership effectiveness can influence and build an organizational culture in the workplace. Alimudin et al. (2017) showed the influence of spiritual leadership on organizational culture and stated that values that have long been considered spiritual idealization, such as integrity, honesty, and simplicity, are almost always present as ineffective leadership practices. Integrity and honesty are the credible dimensions of leadership.

The study results were reinforced by the average value of respondents’ responses to high credible leadership style (3.83), followed by the average value of respondents’ responses to organizational culture measurements (3.97). The results are in line with some previous empirical studies (Hosseini et al., 2020; Gholamzadeh et al., 2014), which show a significant positive influence of leadership styles seen from transformational leadership styles and transactional leadership styles on organizational culture. Respondents in this study were primarily women. This is indicated to be related to the magnitude of the influence of credible leadership on organizational culture. Some results of previous research studies (Herrera et al., 2012; Kawatra and Krishnan, 2014) found the impact of gender and leadership on organizational culture. Most female employees are delighted with credible leadership practices because they promote integrity, honesty, and vision, provide opportunities to participate, are capable, and inspire them to follow the concrete actions shown by their leaders. The greater the number of women in the organization, the more likely it is to prefer participatory leadership; the less likely it is to prefer self-protective leadership; and the more likely organizations to prefer gender egalitarianism (Herrera et al., 2012). This follows the results of the previous study (Brown and Johnson, 2003) mentioned that gender affects leadership styles, and leadership styles impact organizational culture.

### Influence of organizational culture on work engagement

The test results of the influence of organizational culture on work engagement showed significant positive results. The organizational culture that is consistently applied can strengthen work engagement. Organizational culture connects organizational norms, beliefs, values, and principles with its employees, and those assumptions are included in them as a set of standard behaviors and activities (Soni, 2019), one of which is work engagement. Work engagement is a direct result of a strong corporate culture. It refers to how employees feel about their culture and work. The stronger an organization’s culture, the better employees understand what is expected and what they are working on. Engaged employees are more likely to remain happy, motivated, and committed to work and the company.

The response of respondents also supported the results of this test; the majority of women on the characteristics of the organizational culture studied obtained a high average score (3.97), followed by a high average work engagement score (4.18). The results of this study are related to the majority of respondents being women. In Indonesia, the role of women in the world of work is increasing. However, traditionally, as a woman, the gender role inherent in her, she must prioritize family affairs even though women are currently working. If a person can manage several roles simultaneously, those roles will enrich his life. For example, a woman who works as a nurse and carries out health services to others is not an easy job because of her needs or personal interests, and the family may have to be prioritized.

However, on the other hand, there is an improvement in the quality of life in carrying out roles in work or family, which theoretically can increase positive energy and emotions in carrying out duties as a nurse or wife and mother in the family, where positive energy and emotions that arise in the workplace are indications of work engagement. This condition will arise if the organizational culture supports the female workforce. For example, leaders can be role models, respect the dual role of female workers, act fairly, trust, open, organizational justice, good cooperation in the team, and provide awards based on individual and group work results. Rastogi and Saikia (2019) mentioned that most women nurses show that positive supervisors’ positive behavior can improve hospital health services by increasing nurse engagement in very complex, negative, and uncertain hospital environments. If an organization has a strong culture, it will affect the high level of employee engagement in an organization (Kotrba, 2010). This indicates that the stronger the culture, the more influencing the behavior of members. This high similarity and intensity can build cohesiveness, loyalty, and commitment to the organization. Aspects of organizational culture that emphasize integrity, professionalism, praiseworthy, and adequate recognition for human resources can impact employee engagement. The analysis results follow previous research studies, which show that work culture positively influences work engagement (Bija, 2020; Nurcholis and Budi, 2019); organizational culture significantly positively influences work engagement (Siddique, 2019).

### The influence of credible leadership on work engagement with organizational culture as mediation

In the indirect test, credible leadership on work engagement through organizational culture requires credible leadership as the independent variable that significantly influences organizational culture as mediating and organizational culture. The latter significantly influences work engagement. In the indirect test, credible leadership on work engagement through organizational culture requires credible leadership as the independent variable. The test results (Table 5) show that the mediation influences testing requirement and credible leadership has a significant positive influence on organizational culture and work engagement. The role of organizational culture in the influence of credible leadership on work engagement is perfect mediation. The study results show that credible leadership does not necessarily influence work engagement but through the organizational culture formed and influenced by organizational leaders through credible leadership applied by organizational leaders. Nevertheless, other studies have shown that the practices and behaviors of credible leaders appear to have the most impact on engagement (Matthews, 2008).

This is shown in the results of testing that the influence of credible leadership on work engagement becomes insignificant after controlled organizational culture. These results support a previous study by Bija (2020), which showed that work culture mediates the influence of leadership on work engagement. These findings support the results of the study as a development of research by Sürücü and Yeşilada (2017), which examines the influence of charismatic leadership styles on organizational culture; which examines the development of leadership styles from research results; and Martins's research results, Martins (2019), Soni (2019), and Siddique (2019) who examines the influence of organizational culture on work engagement. This suggests that credible leadership indirectly affects the work engagement of medical and non-medical workers in the hospitals that are the object of research, but instead through the internalization of organizational culture. Credible leaders show integrity, respect employee contributions, and are capable in managerial and visionary roles. Subordinates trust them, and subordinates are more committed and provide time, energy, intelligence, and organizational support through the role of subordinates.

## Conclusion

Credible leadership can significantly improve the organizational culture, so credible leadership needs to be strengthened and further developed in the organizational environment to encourage the formation of a strong corporate culture. The authors consider several implications for further studies of hospital leadership practices to enhance a strong organizational culture for increased work engagement. The results of this study show that leaders play an important role in achieving organizational change and hospital development. Hospital management needs to emphasize and develop credible behavior in every leader in every division in the hospital.

The organizational culture can significantly increase work engagement. Hospital management can help their human resources better internalize the values of a superior culture in hospitals in their respective roles, so attachment to work and organization is getting higher. The work engagement of medical personnel, especially nurses, is critical to pay attention to the hospital management so that the performance of hospital services and the satisfaction of patients and patient's families are further improved. It is supported by Gallup’s study of more than 200 hospitals, which showed that nurse engagement rates were the number one predictor of patient mortality rates (https://news.gallup.com/businessjournal/182195/hospitals-performance-management-improved-fast.aspx). By having medical personnel and non-medical personnel more enthusiastic (vigor) when carrying out their duties and responsibilities, becoming more dedicated to patient service duties (dedication), and happy with their profession even though they have to work hard (absorption).

The role of organizational culture mediation on the influence of credible leadership on work engagement is the perfect mediation. Work engagement is a change in the organization’s culture through changes in how leaders lead the organization, and credible leadership is an effective practice in achieving a competitive advantage in service to consumers.

### Limitations

This study has some limitations. First, all data is collected using self-report questionnaires that increase the likelihood of responses being affected by such collection methods. Second, the research sample came from 2 hospitals in Madiun City, and there were 3 hospitals with other types C that had not been studied. For the development of further research, sampling can be carried out at type C hospitals that have not been surveyed. Other recommendations for further research, studies with similar themes, especially credible leadership, can be done in other organizations, or further research can conduct studies of other leadership styles such as integrative leadership. In this study, only assessing credible leadership and organizational culture about work engagement, further research that wished to research with similar objects was advised to include other factors that could influence work engagement, e.g., work–family enrichment. Another recommendation is that the measurement of organizational culture can use other dimensions, for example, clan culture, adhocracy culture, hierarchical culture, and market culture (Mushtaque and Siddiqui, 2020). Further research is carried out by adding employee loyalty and employee retention as a result of work engagement.

## Supplementary information

Ethical clearance

Supplementary Material
