# Supplementary material for: Organizational culture as a mediator of credible leadership influence on work engagement: empirical studies in private hospitals in East Java, Indonesia
Source: Humanit Soc Sci Commun. 2022 Aug 17;9(1):274. doi: 10.1057/s41599-022-01289-z (PMC9381403; doi:10.1057/s41599-022-01289-z)
Supplement: Supplementary file 1 — Ethical clearance [file 41599_2022_1289_MOESM1_ESM.pdf]

PUSAT PENGELOLA JURNAL UNIVERSITAS DR. SOETOMO
Centre of Scientific Journal and Publication - Dr. Soetomo University
Jalan Semolowaru 84 Surabaya, 60118 Telp. (031)5925970 Fax.(031)5938935
Email: ejournal@unitomo.ac.id, Website: ejournal.unitomo.ac.id

Yustinus Budi Hermanto
Universitas Katolik Darma Cendika

ETHICAL CLEARANCE STATEMENT
Ref: 125/K/PPJ/III/2022

Reference is made to the above heading.

I am pleased to inform you that chairman of the Centre of Scientific Journal and Publication Dr Soetomo University, approved ethical clearance of the research titled:

Organizational culture as a mediator of credible leadership influence on work engagement: empirical Studies in private hospitals in East Java, Indonesia

Conducted by: Yustinus Budi Hermanto, Veronika Agustini Srimulyani

Based on recommendation of Commission of Publication PPJ Dr Soetomo University held on 2 Februari 2022 led by Dr. Nurannafi F .S. Maela, S.I.Kom., M.I.Kom.

The validity of this Ethical clearance is one year effective from 11 March 2022 to 11 March 2023

Surabaya, 11 March 2022

Secretary,

Anik Vega Vitianingsih

Cc:
1. VP of Research and International Partnership
2. Rector of Universitas Katolik Darma Cendika
