# Supplementary material for: Organizational culture as a mediator of credible leadership influence on work engagement: empirical studies in private hospitals in East Java, Indonesia
Source: Humanit Soc Sci Commun. 2022 Aug 17;9(1):274. doi: 10.1057/s41599-022-01289-z (PMC9381403; doi:10.1057/s41599-022-01289-z)
Supplement: Supplementary file 2 — Supplementary Material [file 41599_2022_1289_MOESM2_ESM.docx]

**Appendix**

1. **Credible Leadership**

| No | Statement |
| --- | --- |
| 1 | My leader has always treated me honestly and respectfully |
| 2 | My leader admits his mistakes when he makes mistakes |
| 3 | My leader does not hide important information from himself |
| 4 | My leader behaves with his power in order to make me obedient |
| 5 | My leader thinks all employees have adequate abilities to carry out tasks independently |
| 6 | My leader is confident when making decisions |
| 7 | My leader always watches me while doing work |
| 8 | My leader tolerates my job |
| 9 | My leader is always on the lookout when someone else drops |
| 10 | The leader wants me to do as the leader orders |
| 11 | My leader always faces problems and solves them head-on |
| 12 | My leader set an example of behaving well to subordinates |
| 13 | My leaders enjoy discussing with regards to the strengths and weaknesses of the self |
| 14 | My leader gave me the opportunity to learn and develop competence in work. |
| 15 | My leader set a real goal for this hospital |
| 16 | My leader clearly conveyed the goals that this hospital is trying to achieve |
| 17 | My leader has a real vision and mission for this hospital |
| 18 | My leaders kindly share and communicate the vision and mission with subordinates |
| 19 | My leader makes an objective assessment of the resources in the division he/she leads |
| 20 | My leaders are passionate about the vision and mission of this hospital |
| 21 | My leader has always tried in the interest of achieving the goals of this hospital. |
| 22 | My leaders have high principles and commitment in working |
| 23 | My leader has always cared about subordinates |
| 24 | My leader always improves work coordination to make it more effective. |
| 25 | My leader gives directions on how to do a good job |
| 26 | My leader shares complete information about decisions, plans, and activities related to my field of assignment, in order to be able to do the job effectively |
| 27 | My leader collects information regarding work activity and external conditions affecting the work of subordinates |
| 28 | The leader encouraged me with helpful suggestions to be better at work. |
| 29 | My leader gave me adequate responsibility and discretion in the performance of my job |
| 30 | My leaders provide sympathy and support if I experience confusion and anxiety while carrying out my job. |
| 31 | My leaders provide training and career advice that helps me be better at work. |
| 32 | My leaders make it easy for me to solve conflicts, encourage teamwork and identify problems together. |
| 33 | My leader invites subordinates to socialize informally with others. |
| 34 | My leader gives praise and recognition to the performance of subordinates |
| 35 | My leader rewards subordinates for successfully meeting the standards in their assigned work |

**B. Organizational Culture**

| No | Statement |
| --- | --- |
| 2 | I always try to follow the rules that have been set by this hospital |
| 3 | I am always responsible for carrying out my duty role in this hospital |
| 4 | I always strive to carry out my duty role effectively |
| 5 | I carry out my duty role with discipline |
| 6 | I acted fairly in carrying out my duty role in this hospital |
| 7 | I am being firm in carrying out my duty role in this hospital |
| 8 | I was given a self-development program by this hospital. |
| 9 | The management of this hospital treated me based on mutual trust |
| 10 | I and other employees respect each other |
| 11 | I work closely with other co-workers in fulfilling tasks |
| 12 | I was awarded based on the results of my performance |
| 13 | I was awarded based on the results of the group's performance. |

**C. Work Engagement**

| No | Statement |
| --- | --- |
| 1 | At my work, I feel bursting with energy |
| 2 | I find the work that I do full of meaning and purpose |
| 3 | Time flies when I am working |
| 4 | At my job, I feel strong and vigorous |
| 5 | I am enthusiastic about my job |
| 6 | When I am working, I forget everything else around me |
| 7 | My job inspires me |
| 8 | When I get up in the morning, I feel like going to work |
| 9 | I feel happy when I am working intensely |
| 10 | I am proud of the work that I do |
| 11 | I am immersed in my work |
| 12 | I can continue working for very long periods at a time |
| 13 | To me, my job is challenging |
| 14 | I get carried when I am working |
| 15 | At my job, I am very resilient mentally |
| 16 | It is difficult to detach myself from my job |
| 17 | At my work, I always persevere, even when things do not go well |
